# Health equity monitoring is essential in public health: lessons from Mozambique

**DOI:** 10.1186/s12992-019-0508-4

**Published:** 2019-12-18

**Authors:** Alba Llop-Gironés, Lucinda Cash-Gibson, Sergio Chicumbe, Francesc Alvarez, Ivan Zahinos, Elisio Mazive, Joan Benach

**Affiliations:** 1 Health Inequalities Research Group-Employment Conditions Network (GREDS-EMCONET), Universitat Pompeu Fabra, Barcelona, Spain; 2 Johns Hopkins University University Pompeu Fabra Public Policy Center, Barcelona, Spain; 3 GREDS-EMCONET, Department of Political and Social Sciences, Universitat Pompeu Fabra, Ramon Trias Fargas 25-27, 08003 Barcelona, Spain; 4 National Institute of Health, Ministry of Health of Mozambique, Maputo, Mozambique; 5 Instituto Nacional de Saude, Eduardo Mondlane Ave, 1008 Maputo, Mozambique; 6 Medicus Mundi Mediterrània, Secretari Coloma st 112, 08024 Barcelona, Spain; 7 National Institute of Statistics of Mozambique, Maputo, Mozambique; 8 Instituto Nacional de Estatística, 24 de Julho Ave, 1989 Maputo, Mozambique; 9 Grupo de Investigación Transdisciplinar sobre Transiciones Socioecológicas (GinTRANS2), Universidad Autónoma de Madrid, Madrid, Spain

**Keywords:** Health information systems, Health equity, Public health, Social determinants of health, Sustainable development goals

## Abstract

**Background:**

Countries must be able to describe and monitor their populations health and well-being needs in an attempt to understand and address them. The Sustainable Development Goals (SDGs) have re-emphasized the need to invest in comprehensive health information systems to monitor progress towards health equity; however, knowledge on the capacity of health information systems to be able do this, particularly in low-income countries, remains very limited. As a case study, we aimed to evaluate the current capacity of the national health information systems in Mozambique, and the available indicators to monitor health inequalities, in line with SDG 3 (Good Health and Well Being for All at All Ages).

**Methods:**

A data source mapping of the health information system in Mozambique was conducted. We followed the World Health Organization’s methodology of assessing data sources to evaluate the information available for every equity stratifier using a three-point scale: 1 - information is available, 2 - need for more information, and 3 - an information gap. Also, for each indicator we estimated the national average inequality score.

**Results:**

Eight data sources contain health information to measure and monitor progress towards health equity in line with the 27 SDG3 indicators. Seven indicators bear information with nationally funded data sources, ten with data sources externally funded, and ten indicators either lack information or it does not applicable for the matter of the study. None of the 27 indicators associated with SDG3 can be fully disaggregated by equity stratifiers; they either lack some information (15 indicators) or do not have information at all (nine indicators). The indicators that contain more information are related to maternal and child health.

**Conclusions:**

There are important information gaps in Mozambique’s current national health information system which prevents it from being able to comprehensively measure and monitor health equity. Comprehensive national health information systems are an essential public health need. Significant policy and political challenges must also be addressed to ensure effective interventions and action towards health equity in the country.

## Background

On-going debates about health equity in the context of United Nations (UN)‘s Sustainable Development Goals (SDGs) have re-emphasized the need to invest in comprehensive health information systems (HIS) to enable countries to study the Social Determinants of Health (SDH) and take action on health inequalities, so as to ‘Leave No One Behind’ [1].

The minimum requirements for a comprehensive HIS that can report on the SDH and health equity have already been identified, as well as a list of essential sources of health-related information; these include social stratifiers such as gender, social class, race, ethnicity and place of residence, as well as a diversity of mortality, morbidity and disability outcomes, which include self-assessed physical and mental health [2, 3]. Yet in high- and middle-income countries the best available HIS is limited. In low-income countries (LICs), which face some of the most severe health problems, the picture is much worse, and it is often not clear what kind of health and socio-demographic information is available, or whether the information available can be used to effectively analyse the SDH and monitor health equity [4]. Also, the feasibility of obtaining disaggregated data in LICs to support action at national and local level is often discussed in the scientific literature [5] and international forums, such as the UN Statistical Commission on SDG indicators. However, despite large data limitations, there are examples of data that allow differentiation by social group and geographical area, like that of reproductive, maternal, newborn and child health included in the Demographic and Health Survey (DHS).

Mozambique serves as a useful LIC case study, as it ranks 181st out of 188 countries in the Human Development Index 2016 and is a major recipient of health aid [6] that has large within-country health inequalities evaluated using household budget survey data [7]. The Constitution endows all citizens with the right to health (art. 89), yet the focus of national health policies is mainly on the provision of medical care.

The HIS in Mozambique dates back to 1976, when a system for the registration of preventive, promotional and curative activities was formally established. In the early 1980s, the Ministry of Health (MoH) established a data collection mechanism for all public health facilities based on an annual survey-type form that included some epidemiological indicators. In the 1990s, the HIS was introduced nationwide in the form that is still maintained as the base of the health statistical information today. In 2016, the MoH introduced an Information System for Monitoring and Evaluation (SIS-MA), essentially the electronic equivalent of all paper-based data summaries previously implemented in the HIS. SIS-MA aggregates and reports routine information at the health facility level. The information flux is transferred monthly from the health facilities to the District Services of Health, Women and Social Work (SDSMAS in Portuguese), and towards the Provincial Health Directorate (DPS in Portuguese) and the MoH.

The current HIS is composed of various database systems from different MoH departments, for example, routine information reporting (formerly known as “basic module” and currently SIS-MA), aggregated hospital service delivery data (SIS-H), and the registration of vital events from hospital-based births and deaths (SIS-ROH). Besides this, there are parallel database systems managed by vertical health programs such as Electronic Patient Tracking System (EPTS), a longitudinal information system for HIV patients, the medicines and medical commodities warehouse information system, and the human resources health information system (eSIP). Additionally, it contains population surveys, such as DHS among others.

In the MoH, a Planning and Cooperation Directorate (DPC) is responsible for long-term planning, statistical collection, and the analysis of health data. The Health Information Department, another directorate, is in charge of the management of the HIS and guaranteeing, where possible, the interoperability of the different databases.

Users of the HIS include a number of non-governmental organizations supporting vertical programs, the World Health Organization (WHO) and other UN organizations, the Health Directorates of the MoH such as Public Health, Medical Care, and Planning and Cooperation, the National Health Instituto (INS), and the few national research centers, academic institutions and individual researchers.

The country’s research practices are similar to other post-colonial and resource-dependent countries in Sub-Saharan Africa [8], with strong dependencies on top-down policies, limited governmental support, and with research funding almost exclusively provided by foreign donors and international agencies.

In most LICs, two main types of HIS data sources are found [9]: *institution-based data*, which contain information on people who have interacted with a given institution and *population-based data*, such as household surveys that have information on a representative sample of the population, including census and vital registration that have information on every individual. These two main sources of health and well-being information, while providing useful data, lack up-to-date key population indicators for comprehensive equity-oriented decision-making. For example, poor vital registration systems mean that a large proportion of people are born and die uncounted for, and the reasons for their deaths are largely unknown [10]. Additionally, indicator discrepancies often arise, due to different data collection methods [3], and data quality [11].

Household surveys, such as the DHS, usually have some of the required information to be able to monitor the SDH and the progress towards achieving health equity (e.g. they collect data on health status, demographics and living conditions), yet they are intermittent and limited in power for district-level estimates [5]. Household surveys are typically used by governments in LICs to monitor population health needs, however, as research funding in many LIC is predominantly provided by foreign donors and international agencies, household surveys are also used by donors to tie this information to reporting requirements and programme-specific monitoring, such as maternal and child health or populations at risk of HIV [9].

Over the last two decades, and particularly in line recommendations of the final report of the WHO’s Commission on SDH in 2008, additional efforts have been made to support the mapping of national HIS capacities, the integration of a health equity lens into HIS [12], and the creation of observatories to measure health inequalities in low and middle income countries [13]. Yet a great number of challenges still exist globally, in terms of developing valid and complete sources of information, integrating sectors, linking health databases, embedding infrastructure at the institutional level, as well as gathering, analysing and interpreting data in the local context, and disseminating results to a diverse audience [13, 14].

Knowledge on the progress of national HIS to measure and monitor health equity in LIC, in particular, remains very limited. In response to this knowledge gap, we evaluate the national indicators available in Mozambique to monitor progress towards health equity in line with SDG3 on health and well-being.

## Methods

We mapped the capacity of the HIS of Mozambique to measure progress towards health equity, in line with SDG3. Data source mapping involves cataloguing and describing all available institution- and population-based data sources for a given country to determine which sources can be used for health inequality monitoring [9].

The mapping consisted of four steps [15]: (1) the selection of the indicators to measure health inequalities - in this case we selected the 27 SDG3 indicators; (2) the identification and selection of relevant data sources available from the HIS, which included national-level data from institution-based and population-based data sources. Furthermore, to establish an overview of the capacity and trends in the investment in data in Mozambique, for each relevant data source identified we also noted its funding source (i.e. national or external), and the time period of new available information; (3) an assessment of each data source to evaluate whether, and what type, of information was available for the main equity stratifier (i.e. socio-economic position, education, sex, age, place of residence and race/ethnic group), using a three-point scale [9]; One point was given when the information was available, two points if there was a need for more information, and three points in the case of an information gap. Also, for each indicator, we calculated the national average inequality score using the results of the three-point scale for each equity stratifier; (4) the development of a final list compiling all the information obtained.

To ensure quality control of the study, the selection of databases followed a rigorous screening process, which involved a number of authors. The first author was responsible for screening and charting the main available information, and this was distributed among the authors from the MoH of Mozambique and the National Institute of Statistics of Mozambique to reach consensus on scoring the indicators. The final document was agreed on by the research team.

## Results

Table 1 provides an overview of the data sources, periodicity and funding of the available information to report on each of the 27 SDG3 indicators in Mozambique. There are eight identified data sources: 1) four institution-based sources consisting of SIS-MA, the electronic Personnel Information System for health (eSIP), Hospital Module and, Spectrum; and, 2) four population-based sources: DHS; Household Budget Survey; Vaccination, Malaria and AIDS Indicator Survey (IMASIDA in Portuguese); and, Survey of chronic disease risk factors (STEPS in Portuguese). The time period of the available information in most of the indicators (i.e. nine indicators) is 5 years, with seven of the indicators providing information every year, and one indicator that updates information on a weekly basis. As part of the data source mapping process, a mix of national and external-funded data sources were identified, specifically, seven indicators bear information with nationally funded data sources, ten with data sources externally funded, and ten indicators either lack information or it does not applicable for the matter of the study.

**Table 1:** Data sources, periodicity and funding of the information to report on the indicators to measure SDG3 in Mozambique

Indicators of the SDG 3: Ensure healthy lives and promote wellbeing for all at all ages	Data sources	Time period	Funding
3.1.1 Maternal mortality ratio	DHS	Five-year	External
3.1.2 Proportion of births attended by skilled health personnel	IMASIDA	Five-year	External
3.2.1 Under-five mortality rate	DHS	Five-year	External
3.2.2 Neonatal mortality rate	DHS	Five-year	External
3.3.1 Number of new HIV infections per 1000 uninfected population, by sex, age and key populations	SPECTRUM	Yearly	External
3.3.2 Tuberculosis incidence per 100,000 population	SIS-MA	Yearly	National
3.3.3 Malaria incidence per 1000 population	SIS-MA	Weekly	National
3.3.4 Hepatitis B incidence per 100,000 population	SIS-MA	Yearly	National
3.3.5 Number of people requiring interventions against neglected tropical diseases	Not available	–	–
3.4.1 Mortality rate attributed to cardiovascular disease, cancer, diabetes or chronic respiratory disease	Hospital Module	Yearly	National
3.4.2 Suicide mortality rate	Not available	–	–
3.5.1 Coverage of treatment interventions (pharmacological, psychosocial and rehabilitation and aftercare services) for substance use disorders	Not available	–	–
3.5.2 Harmful use of alcohol, defined according to the national context as alcohol per capita consumption (aged 15 years and older) within a calendar year in litres of pure alcohol	STEPS	Five-year	External
3.6.1 Death rate due to road traffic injuries	Not available	–	–
3.7.1 Proportion of women of reproductive age (aged 15–49 years) who have their need for family planning satisfied with modern methods	SIS-MA	Yearly	National
3.7.2 Adolescent birth rate (aged 10–14 years; aged 15–19 years) per 1000 women in that age group	DHS	Five-year	External
3.8.1 Coverage of essential health services	Not available	–	–
3.8.2 Proportion of population with large household expenditures on health as a share of total household expenditure or income	HBS	Five-year	External
3.9.1 Mortality rate attributed to household and ambient air pollution	Not available	–	–
3.9.2 Mortality rate attributed to unsafe water, unsafe sanitation and lack of hygiene (exposure to unsafe Water, Sanitation and Hygiene for All (WASH) services)	HBS	Five-year	External
3.9.3 Mortality rate attributed to unintentional poisoning	Not available	–	–
3.a.1 Age-standardized prevalence of current tobacco use among persons aged 15 years and older	DHS	Five-year	External
3.b.1 Proportion of the target population covered by all vaccines included in their national programme	SIS-MA	Yearly	National
3.b.2 Total net official development assistance to medical research and basic health sectors	Not applicable	–	–
3.b.3 Proportion of health facilities that have a core set of relevant essential medicines available and affordable on a sustainable basis	Not applicable	–	–
3.c.1 Health worker density and distribution	eSIP	Yearly	National
3.d.1 International Health Regulations (IHR) capacity and health emergency preparedness	Not applicable	–	–

Note: Information System for Monitoring and Evaluation (SIS-MA in Portuguese); electronic Personnel Information System for health (eSIP in Portuguese); Demographic and Health Survey (DHS); Household Budget Survey (HBS); Vaccination, Malaria and AIDS Indicator Survey (IMASIDA in Portuguese); Survey of chronic disease risk factors (STEPS in Portuguese)

Results from the data source mapping also show that none of the 27 SDG3 indicators to measure progress on health equity in Mozambique, can be fully disaggregated by an equity stratifier. Currently, the indicators either lack some information (i.e. 15 indicators) or do not have any information at all (i.e. nine indicators). The national average inequality score indicates that those indicators that have more information, are the ones related to maternal and child health (Table 2).

**Table 2:**
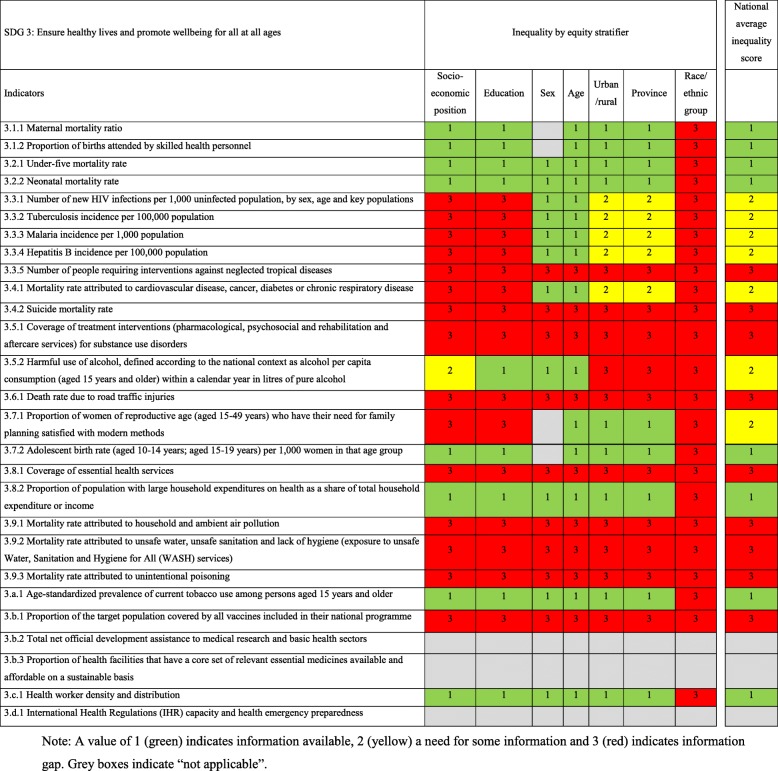
Indicators to measure SDG3 in Mozambique, inequality by equity stratifier and national average inequality score

Note: A value of 1 (green) indicates information available, 2 (yellow) a need for some information and 3 (red) indicates information gap. Grey boxes indicate “not applicable”

Of the total 27 SDG3 indicators, the majority can be disaggregated by sex and age, 15 cannot be stratified by socio-economic position or education, only nine indicators have complete information of the geographical area, and five indicators have gaps in the geographical information. Finally, none of the indicators can be disaggregated by race or ethnicity (Table 2).

## Discussion

The development of a sustainable and effective national HIS that is capable of analysing the SDH and health inequalities, as well as assessing and monitoring action towards health equity, is an essential public health need in every country. Such systems need to be strengthened across all country income groups, particularly in LICs. This study aimed to evaluate the current capacity of the national HIS in Mozambique to monitor health inequalities in line with the 27 indicators of the SDG3 on health and well-being, and potentially provide some insight into the information gaps that might exist in the national HIS of other LIC.

Despite the increased interest and sophistication in monitoring the SDH, as well as the growing acknowledgement of its importance to measure progress towards health equity [16], our results show that in the case of Mozambique, a large proportion of the SDG3 indicators require information that is only available from externally funded data sources, and none of the indicators can be fully disaggregated by the equity stratifiers due to the lack of information on race and ethnicity, since the country does not collect such information. The improvement of HIS is complicated, particularly in LIC setting where there are numerous global health initiatives [17], and the limited public health information that is available in LIC, appears to be partly tied to those who demand certain types of data for specific funding or reporting purposes [3]. For example, our results show that the most complete information to monitor health equity in Mozambique is related to the maternal and child health programmes which are often external-donor-funded programmes.

Studies assessing the national health research systems in Guinea-Bissau, Gambia and Mali have shown that health research in Sub-Saharan Africa depends to a greater extent to external donor funding and top-down policies rather than on domestic support [18–20]. All of this has likely influenced the evolution of national health research training and infrastructure, the nature of health (and health inequalities) research conducted, as well as the use of health (and health inequalities) research findings; collectively, this might have hindered progress in strengthening the capacities of national HIS to integrate a health equity lens, within this context. This might also partly explain why Mozambique has only made a limited contribution to the global scientific production on health inequalities [21].

The SDGs and their ‘Leaving No One Behind’ agenda, have brought a renewed momentum and commitment to strengthening national HIS with a focus on health equity; however, as Palmer et al. note, this promotion should also emphasise the importance of health (and health inequalities) research being internally driven, owned by the country, and in line with identifiable national health and well-being priorities, as well as to ensure that this research feeds into local policy and practice to improve population health and well-being, and address the SDH inequalities, in the context of national development [19].

The introduction of the SIS-MA in 2016 in Mozambique, was a step forward of the MoH towards producing comprehensive and up-to-date routine information of good quality, however, the results of the study show a lack of routine health information able to capture health and mortality trends differentiating social groups and geographical area. These results suggest that there is a need to define a set of determinants and indicators that are issue-specific and match populations health and well-being needs. For example, despite in Sub-Saharan Africa only 24.2% of the population has access to safe water [22], the mortality rate attributed to unsafe water is currently unavailable. Also, it will not be adequate to restrict the indicators whose Mozambique has more information (i.e. maternal and child health), since it only concerns certain population groups, as women of reproductive age and children.

Despite the limitations identified in the national HIS capacity to monitor health equity, Mozambique has recently made steps forward towards aiming to strengthen the capacity of its national HIS to monitor population health and strengthen the intersectoral data linkages. It is currently undergoing a process of strengthening the civil registration and vital statistics (CRVS) – an urgent country need, as the registration rate of children under five has been estimated to be less than 50% [23]. This progress involves different ministries and national institutions such as Ministries of Justice, Constitutional and Religious Affairs, Ministry of Health, National Institute of Statistics and Ministry of Interior; in addition to international partners (i.e. WHO and UNICEF) and donors (i.e. the government of Canada). Currently, they are focusing on the legislative and policy framework, the registration of all vital events, and the interoperability between data management systems.

Data source mapping enables the critical review of a range of data sources and can embed an equity lens in the appraising of HIS, not only at the national level, but at lower administrative levels if desired [9]. However, it should be noted that data source mapping is limited in the appraisal of the quality of the data, and it lacks capacity to capture information fluxes outside official data sources, such as verbal, observational or written information that could occur at health facility level. Nevertheless, our results identify specific systems gaps that need to be addressed in order to strengthen the measuring and monitoring of health equity at country level, and to our knowledge, this is the first study that has assessed the capacity of the national HIS to monitor progress towards health equity, and SDG3. As such, these study findings could be used to directly inform and support the current action that is underway in Mozambique to strengthen the national HIS.

## Conclusion

Information gaps in the current health information system in Mozambique prevent the country from being able to comprehensively measure and monitor health equity, so as to leave no one behind. A large proportion of the 27 indicators of SDG3, focused on health and well-being, require information that is only available from externally funded data sources, and none of the indicators can be fully disaggregated by the equity stratifiers. The main recommendations towards the development of a sustainable and effective national health information system capable of analysing the social determinants of health will be to define the set of determinants and indicators that are issue-specific and match populations health and well-being needs of the country, as well as strengthen the health sector and intersectoral data linkages. In addition, adequate investment in technical and human resource capacities to address health inequalities is needed, which is likely to be the case in similar settings. To support this, a number of policy and political challenges must also be addressed to support transparency in decision making process and accountability for action. For example, the coexistence of political leadership, together with effective communication and collaboration between Ministries and the National Statistics Offices, and whole-of-government and whole-of society engagement is required. Comprehensive national health information systems are essential public health needs, as their absence not only hides the health equity gap within countries, but also limits national capacity to effectively inform local interventions and action towards achieving health equity.

## Data Availability

The datasets analysed during the current study are available under request to the Ministry of Health of Mozambique.
